# A Contemporary Review of Cryptorchidism Management in Adults: A Rare Presentation of Bilateral Cryptorchidism Presenting as Pelvic Pain in an Adult Patient

**DOI:** 10.7759/cureus.52933

**Published:** 2024-01-25

**Authors:** Colleen B Sholtes, Lauren A Tranthem, Fumihiko Nakamura, Katie Canalichio, Michael Goedde, Kellen Choi

**Affiliations:** 1 Department of Urology, Cleveland Clinic Akron General, Akron, USA; 2 Department of Urology, University of Louisville School of Medicine, Louisville, USA; 3 Department of Pediatric Urology, Norton Children’s Hospital, Louisville, USA; 4 Department of Urology, University of Louisville Hospital, Louisville, USA

**Keywords:** pelvic pain, cryptorchidism, bilateral, adult, udt

## Abstract

This case report presents a rare case of adult cryptorchidism, found incidentally in a 25-year-old gentleman who initially presented with abdominal and suprapubic pain and was successfully treated with staged orchidopexy. To our knowledge, to date, our case is the first published instance of bilateral cryptorchidism in an adult presenting with nonspecific suprapubic pain.

Cryptorchidism is the most common genital abnormality in newborn boys, and due to its association with an increased risk of infertility and malignancy, current management involves surgical correction with orchidopexy by 12 to 18 months of life. Adult presentation of cryptorchidism is very unusual due to early intervention; therefore, bilateral cryptorchidism is even more rare. As a result, current guidelines do not address proper management for adult cryptorchidism.

Therefore, after performing a thorough review of the literature on contemporary guidelines for cryptorchidism management, we aim to highlight our approach to management in this rare case of adult bilateral cryptorchidism. We suggest bilateral orchiectomy as the safest option, if the patient is amendable, or bilateral orchiopexy with long-term follow-up for testicular cancer. Although the American Urological Association guidelines recommend orchiectomy for postpubertal cryptorchid children, currently, no explicit guidelines exist for the preferred method of managing adult cryptorchidism. Due to the increased risk of infertility and testicular cancer with cryptorchidism, orchiectomy instead of orchiopexy may be the preferred surgical approach in some instances. Still, in the case of bilateral cryptorchidism, orchiectomy may not always be the most viable solution, making orchiopexy with long-term follow-up for testicular cancer the best option, such as in our case.

## Introduction

The presentation of cryptorchidism, or undescended testicles (UDTs), in adult males is rare and is exceedingly rarer when presenting bilaterally. Because of this, surgical correction of UDTs is an uncommon procedure among adult urologists, and as a result, no clear-cut guidelines currently exist for the management of cryptorchidism in adulthood.

Cryptorchidism is the most common genital abnormality in newborn boys [1]. Due to the increased risk of infertility and malignancy with cryptorchid testes, current pediatric management involves surgical correction with orchidopexy by 12 to 18 months of life [2].

## Case presentation

Here, we present a rare case of a 25-year-old wheelchair-bound gentleman with cerebral palsy who was incidentally found to have bilateral cryptorchidism during a workup for abdominal pain. The patient was from rural Kentucky with limited access to healthcare, likely contributing to his delayed presentation and workup. The patient had a several-year history of nonspecific lower abdominal pain, left groin pain, and periodic urinary tract infections. He had always been incontinent and unable to void on command and used intermittent self-catheterization to void.

**Figure 1:**
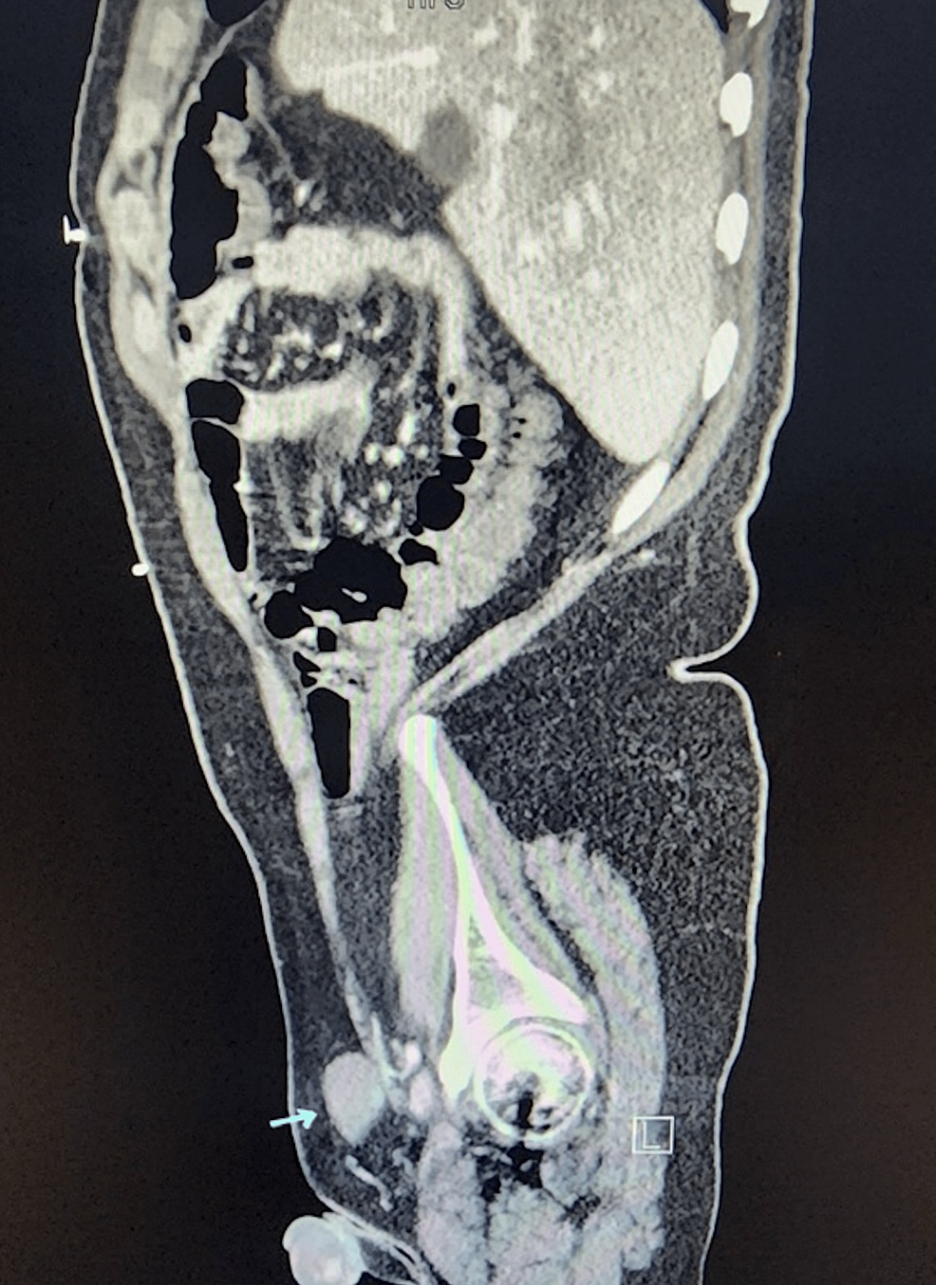
CT scan sagittal view. Blue arrow showing the undescended testicle.

**Figure 2:**
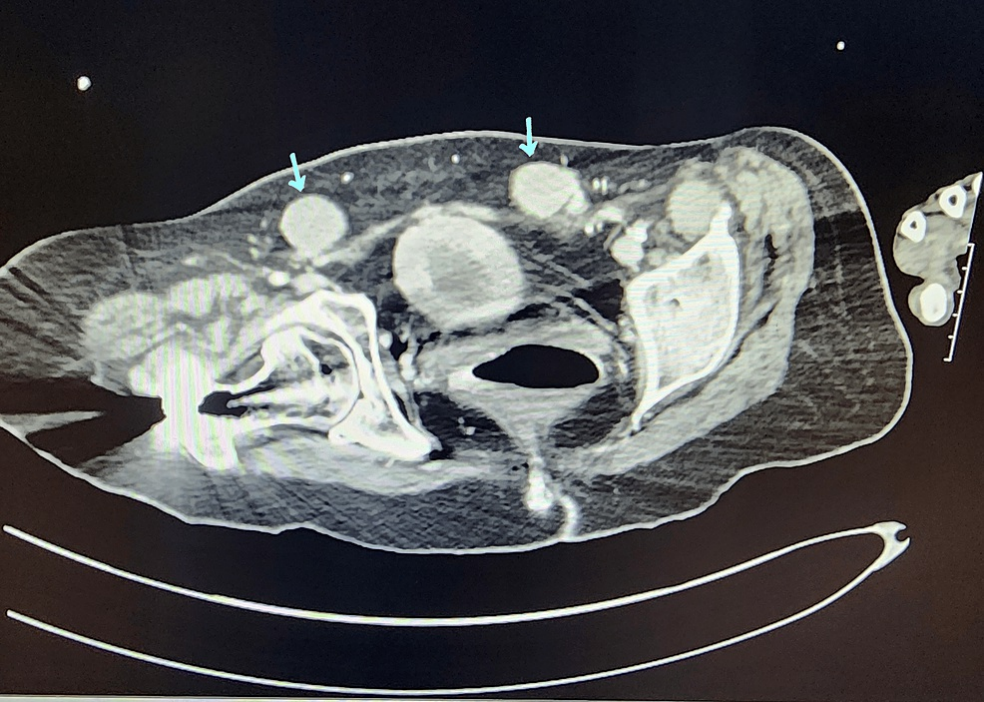
CT scan axial view. Blue arrows showing bilateral undescended testicles.

In February 2021, the patient presented to the emergency department (ED) for abdominal pain. During the workup, a CT scan was done where bilateral UDTs were incidentally found (Figures 1, 2). It was recommended that the patient follow up with urology outpatient.

**Figure 3:**
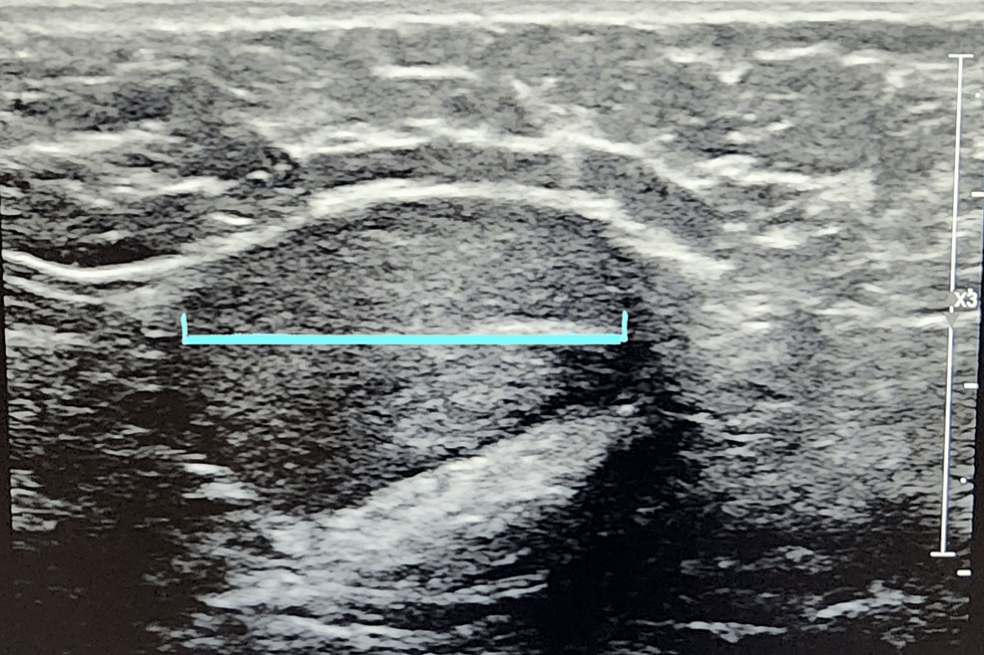
Right inguinal canal with right testicle ultrasound. Blue line showing the length of the right testicle.

**Figure 4:**
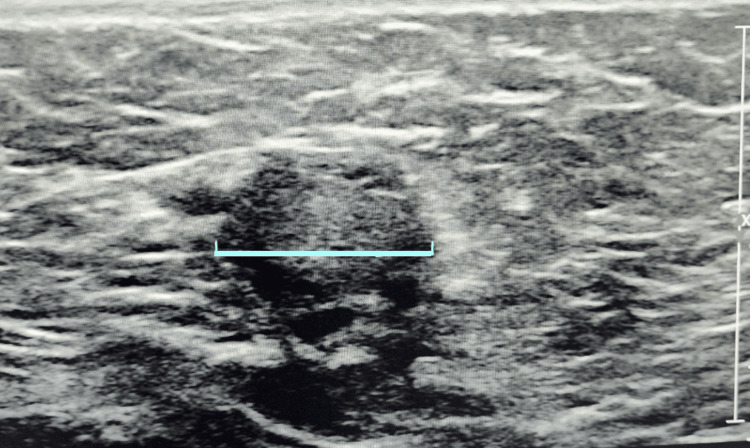
Left testicle ultrasound. Blue line showing the length of the left testicle.

The patient presented to the urology clinic and an appropriate cryptorchidism workup was done. On examination, both testicles were palpable in the inguinal canal. A scrotal ultrasound showed bilateral UDTs in the inguinal canal without associated masses. The testicles were symmetric in size with the right measuring 2.4 x 3.1 x 1.4 cm and the left 3.0 x 2.7 x 1.0 cm (Figures 3, 4). Both testicles demonstrated normal, smooth, oval contour and homogeneous echotexture. There was symmetric color and Doppler intratesticular arterial flow bilaterally. Both left and right epididymis were unremarkable and there were no significant hydroceles, varicoceles, or inguinal hernias.

Due to the increased risk of infertility and testicular malignancy in males with cryptorchidism, especially with an increased duration of time of testes in the cryptorchid position, it was recommended that the patient undergo a bilateral orchidopexy. Although discussed, orchidopexy on one side with a contralateral orchiectomy was an inferior approach in this patient due to the patient’s small size of testes. Removal of one testicle still posed the risk of the patient requiring hormonal replacement, an outcome which we sought to avoid. The patient’s symptoms were mainly left-sided, the same side with an easily palpable testicle. In addition, with the patient’s comorbidities, there was the question of how the patient would respond to invasive surgeries requiring anesthesia. For these reasons, it was decided that the procedure be done in stages with a left orchiopexy done first.

**Figure 5:**
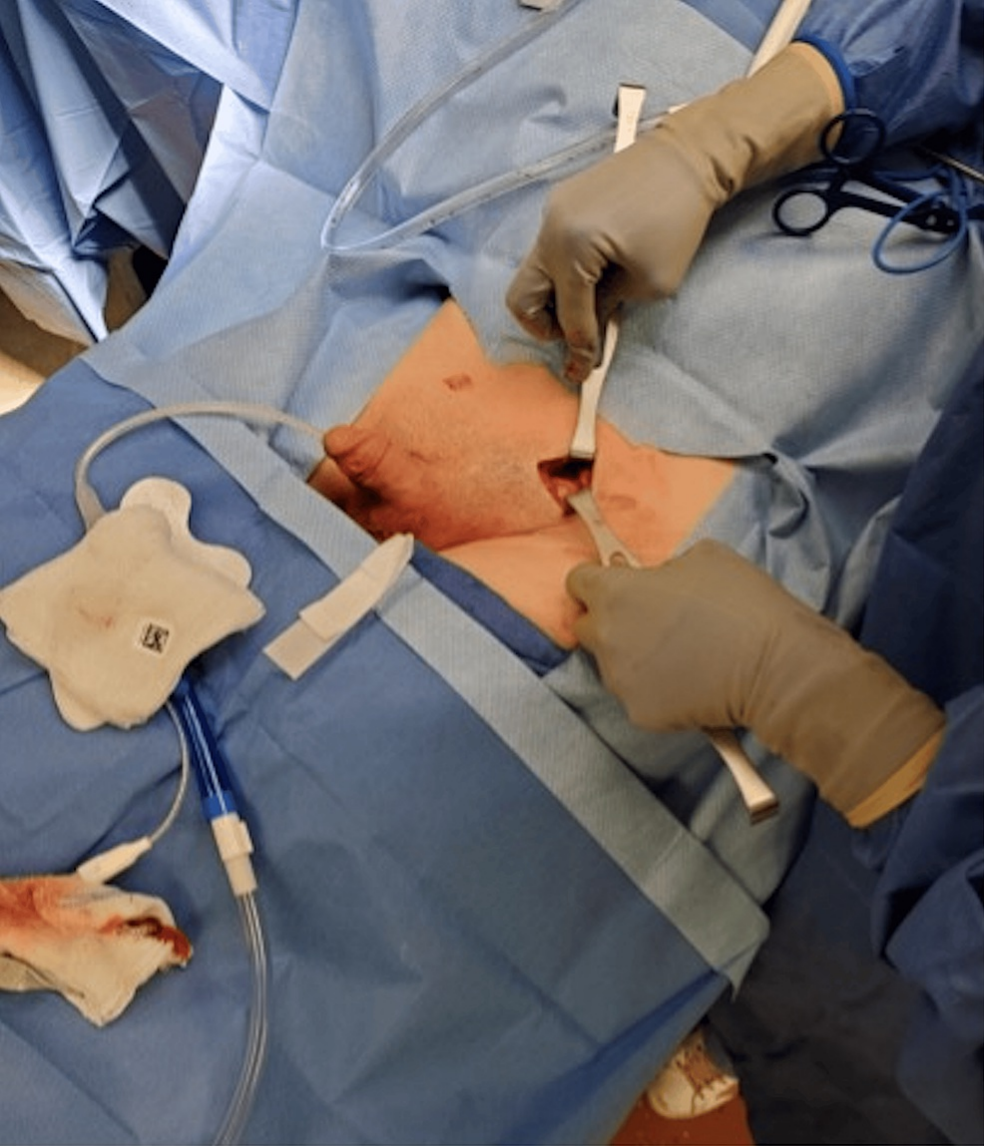
Left inguinal incision.

**Figure 6:**
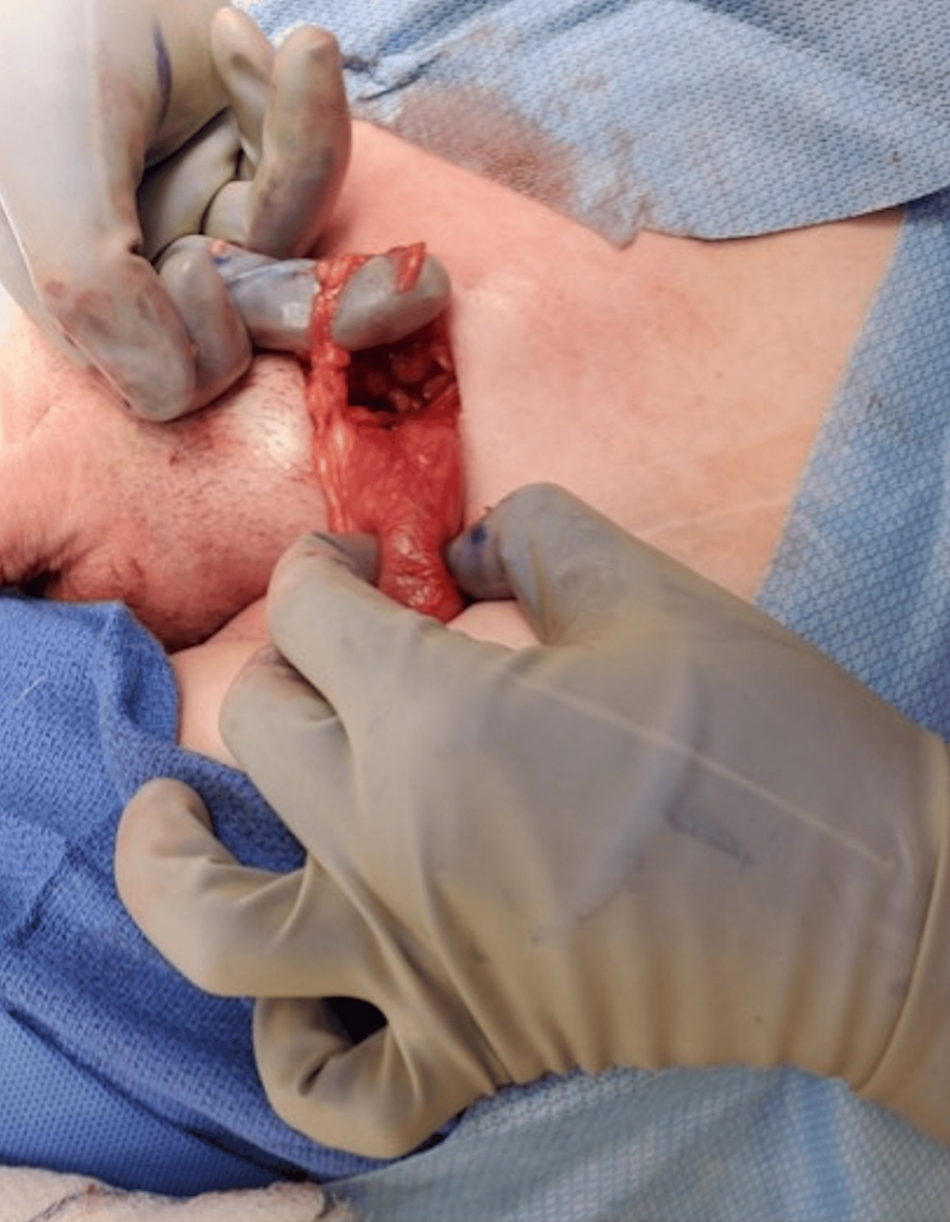
Identification of the left testicle and freeing it from the gubernaculum attachments.

**Figure 7:**
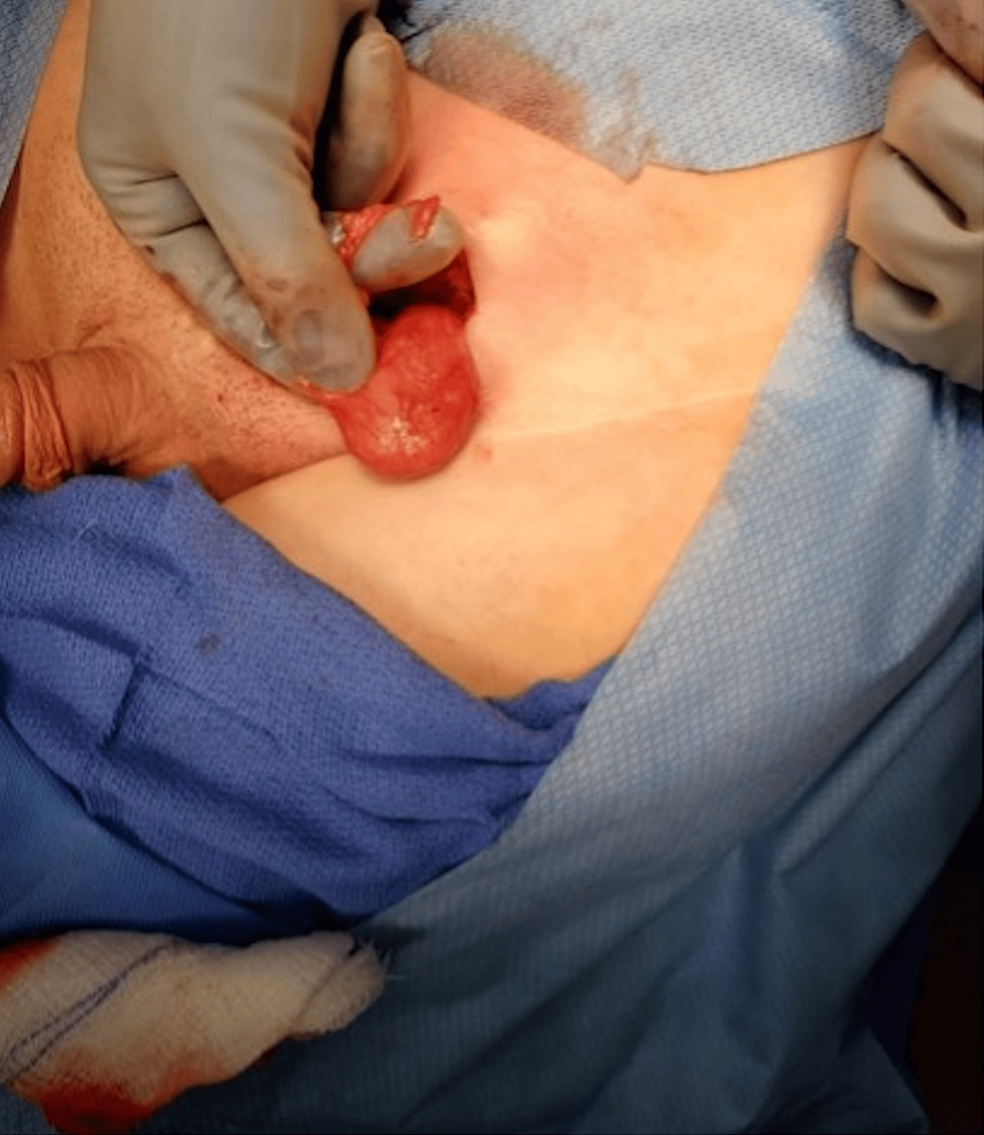
Identification of the left testicle and freeing it from the gubernaculum attachments.

**Figure 8:**
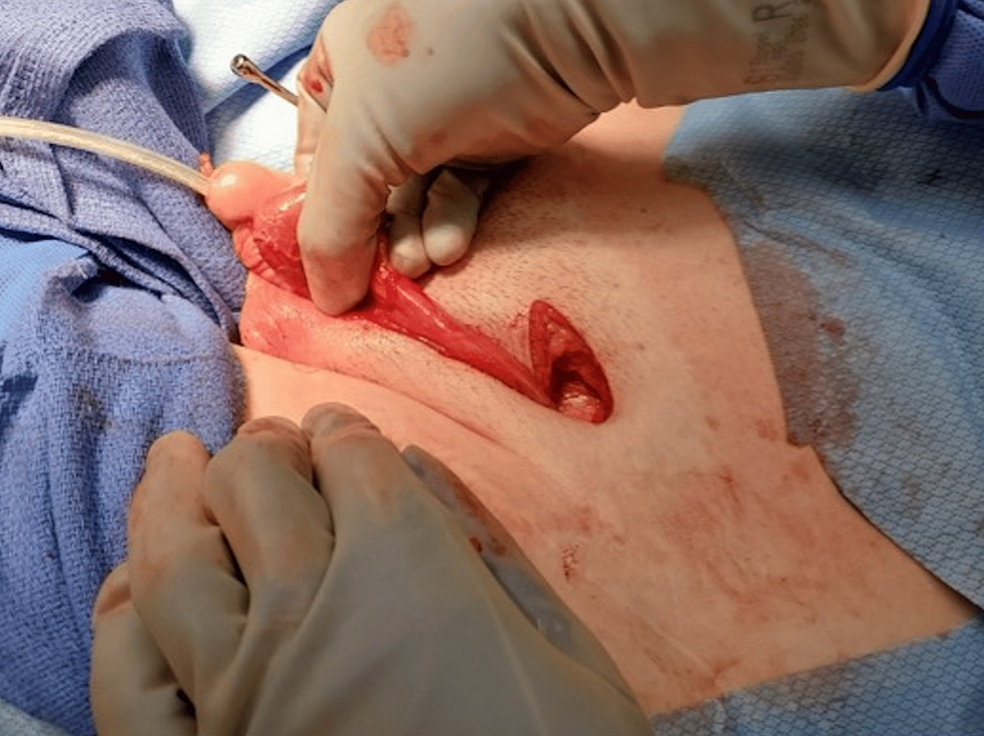
Ensuring good mobility of the spermatic cord.

**Figure 9:**
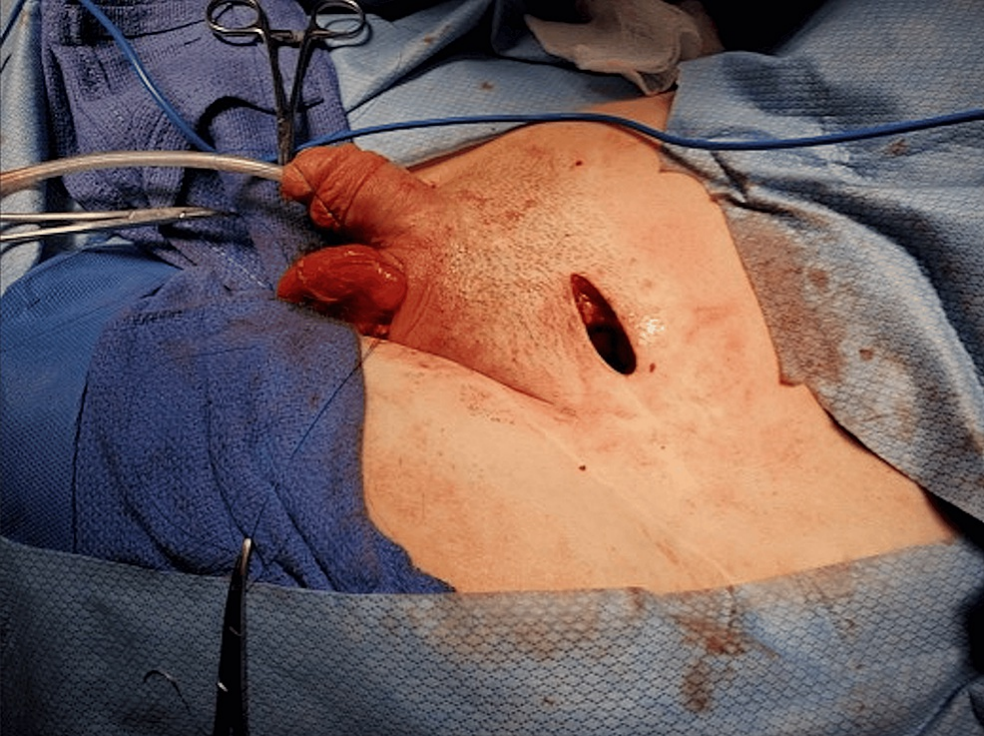
Three-point fixation of the testicle in the scrotum.

Before surgery, the adult urologist performing the procedure consulted a pediatric urology colleague due to the rarity and unfamiliarity of performing such a procedure in the adult population. Left orchidopexy was successfully performed using an inguinal approach. A left inguinal incision was made (Figure 5). Scarpa’s fascia was opened, and the left testicle was easily identified and freed from gubernaculum attachments (Figures 6, 7). The hernia sac was identified and ligated with good mobility of the spermatic cord (Figure 8). Blunt dissection was done from the incision to the left side of the scrotum without difficulty. A sub-dartos pouch was created and a three-point fixation of the testicle in the pouch was done (Figure 9). The scrotum was then closed in three layers.

**Figure 10:**
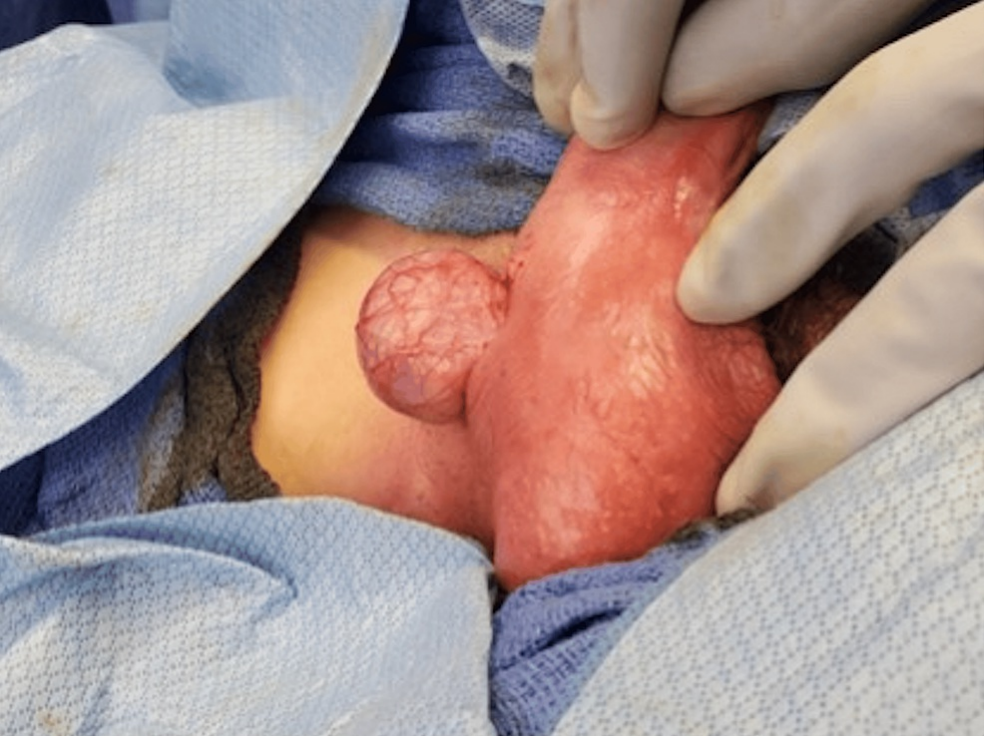
Transverse scrotal incision revealing the right testicle.

**Figure 11:**
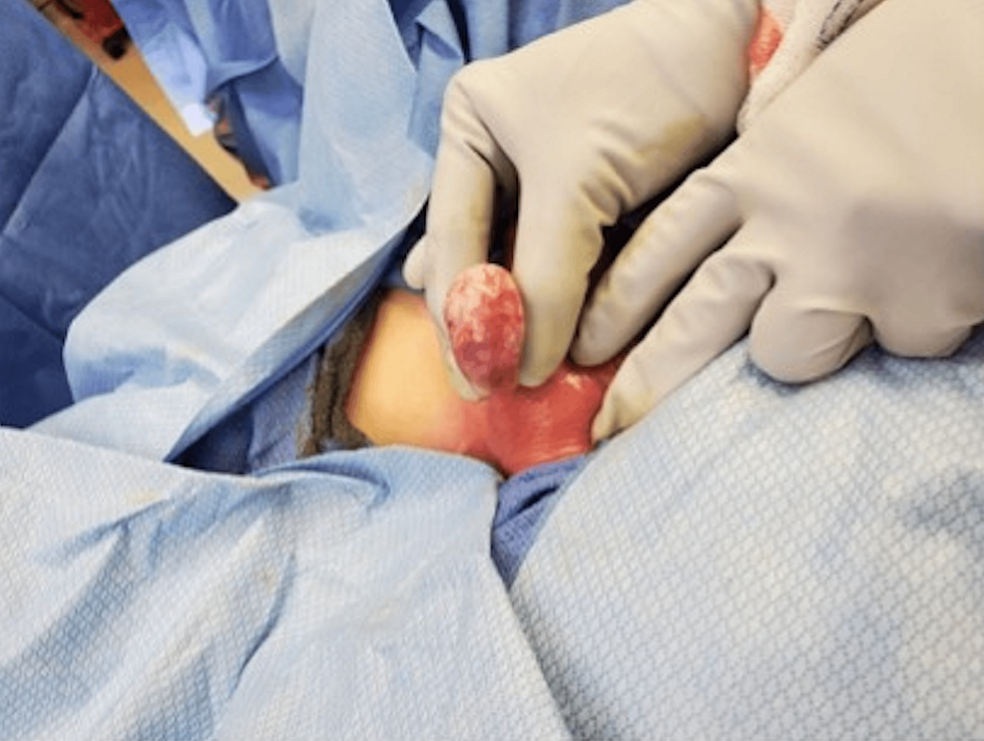
Freeing the right testicle from the gubernaculum attachments.

**Figure 12:**
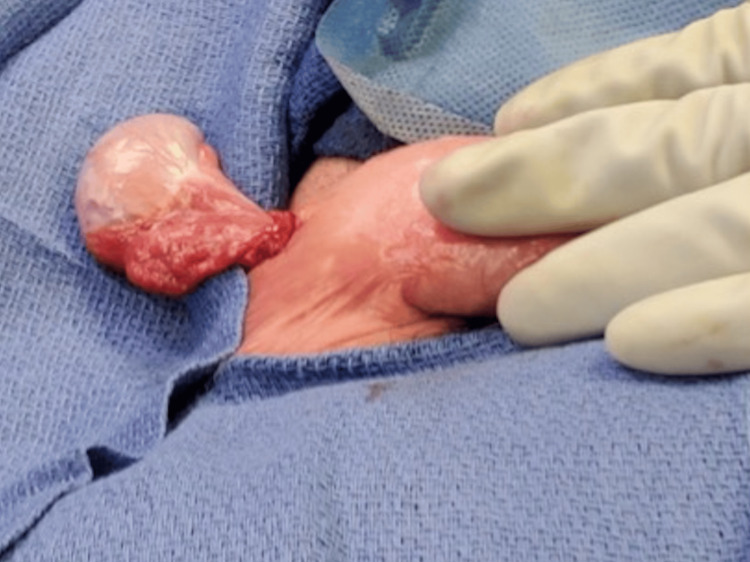
Ensuring good mobility of the spermatic cord.

**Figure 13:**
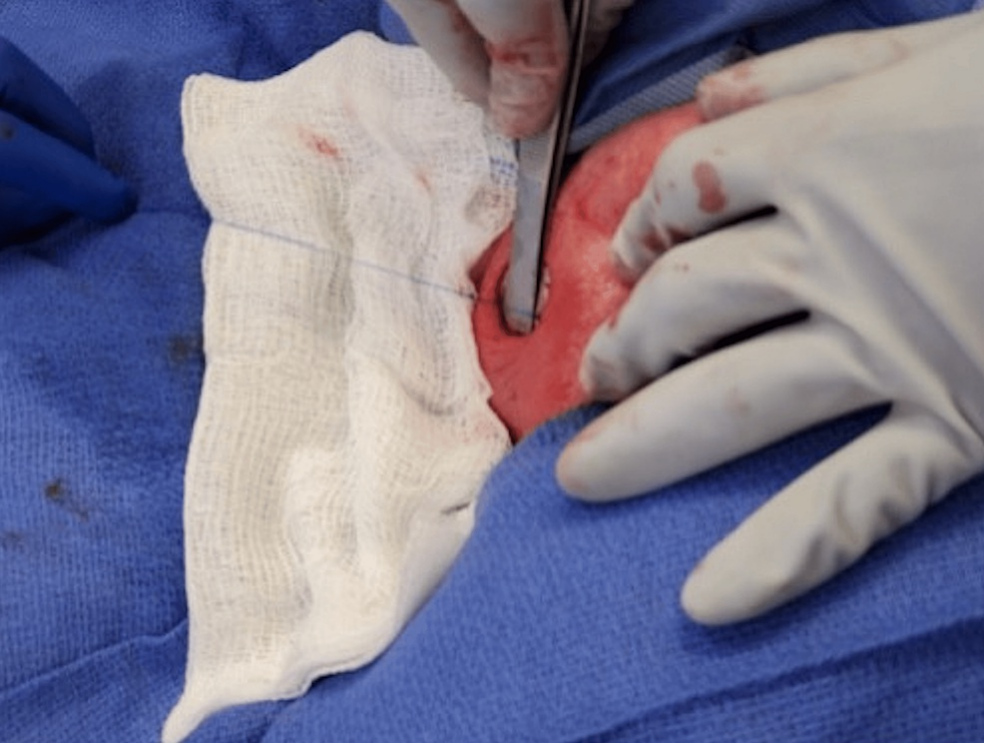
Creating of the sub-dartos pouch.

**Figure 14:**
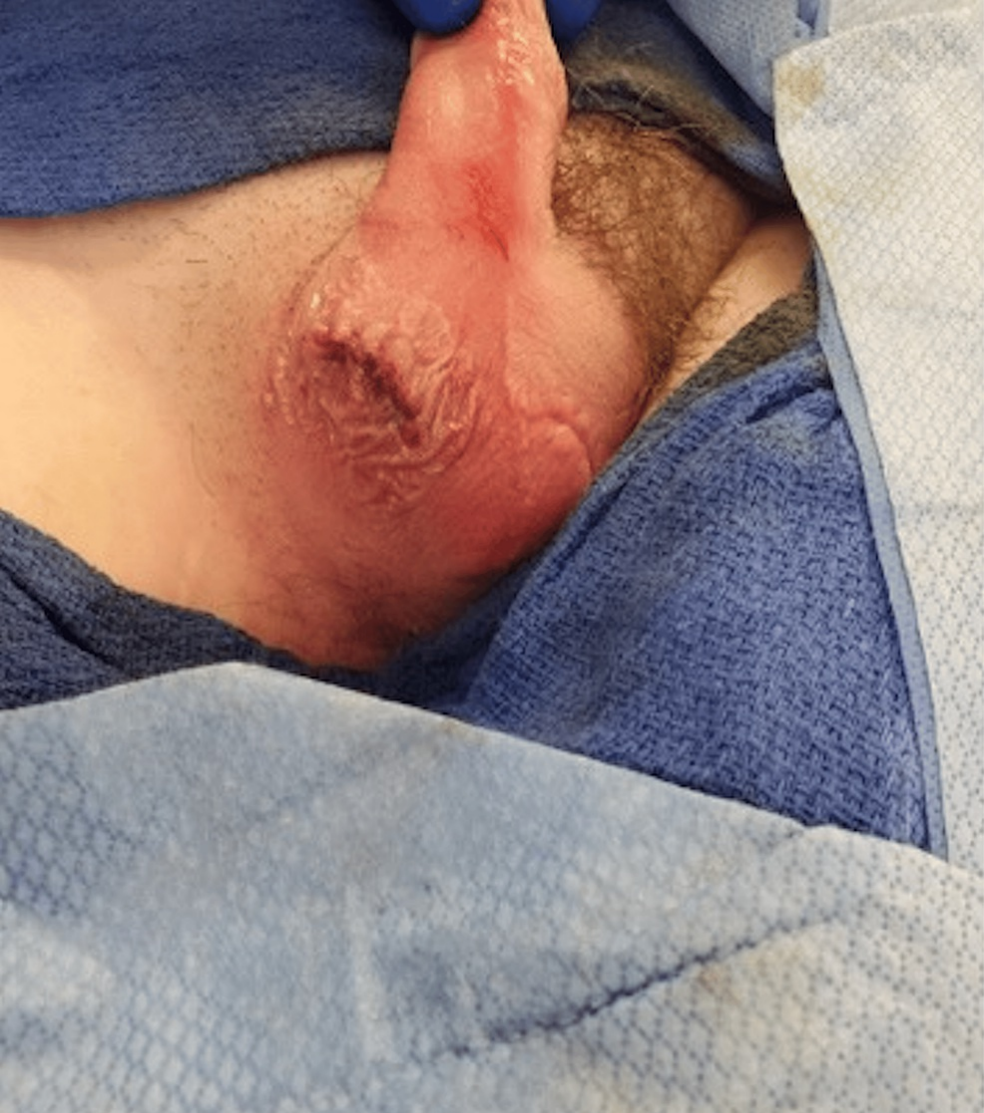
Three-layer closure of the scrotal incision.

Two months later, the right orchidopexy via a scrotal approach was successfully done. At the time of surgery, the left testicle, from the previous orchiopexy, was in a good position in the left scrotum. Once anesthesia was induced, the right testicle was more easily palpated in the scrotum. A transverse scrotal incision was made, immediately revealing the right testicle (Figure 10). Care was taken to avoid crossing the midline where the left orchiopexy was previously performed. The right testicle was freed from gubernaculum attachments, the hernia sac was identified and ligated, and good mobility of the spermatic cord was obtained (Figures 11, 12). Blunt dissection was done from the incision to the right side of the scrotum. A sub-dartos pouch was created with a three-point fixation of the testicle in the pouch (Figure 13). The scrotum was then in three layers (Figure 14).

At the two-week postoperative follow-up, the patient reported markedly improved abdominal and groin pain bilaterally. This suggested his nonspecific abdominal pain was likely due to the incorrect location of the cryptorchid testes.

## Discussion

A PubMed search was done using the terms “bilateral,” “cryptorchidism,” and “adulthood,” and 52 articles were found. We narrowed our search by excluding articles that were published more than 10 years ago, articles that were not in the English language, articles that reported pediatric instead of adult cases, articles that did not specify the age of patients, and articles that were irrelevant to our topic. Seven articles were selected and reviewed [3-9].

Refining the PubMed search further, using the terms “bilateral,” “adult,” “cryptorchidism,” “orchidopexy,” and “orchiectomy,” 35 articles were found. After excluding articles in non-English languages, those that were published over 10 years ago, and those that reported on unilateral cryptorchid cases, pediatric cases, specific surgical techniques, or articles that were irrelevant, five articles were selected and reviewed [10-14].

During the review, the age of surgical intervention, type of surgical intervention (orchidopexy vs. orchidectomy), associated infertility, associated neoplasms, and any other associated abnormality were noted. Of the articles reviewed, there were 10 case reports of adult bilateral cryptorchidism, with an average age of diagnosis and intervention at 31.8 years [3-9,11-13]. Three of the 10 individuals underwent orchidopexy, five individuals underwent orchidectomy, and two underwent orchidopexy on one side with orchidectomy on the other [3-9]. In four of the 10 cases, the cryptorchid testes were located in the inguinal canal, in five cases, cryptorchid testes were located intra-abdominally, and in one case, one was inguinal while the other was intra-abdominal [3-9,11-13]. Seven of the 10 individuals were infertile and all seven were found to have cryptorchid testes on an initial infertility workup [3-9,11-13]. Four individuals had a testicular neoplasm present at the time of surgery, three had abnormal but not neoplastic testicular histology at the time of surgery, and the remaining three had normal histology at the time of surgery [3-9,11-13]. Two individuals had an associated hernia, and one individual was HIV positive [3-9].

Also reviewed was one meta-analysis by Muncey et al. including data from 157 men with bilateral cryptorchidism, all of whom reported preoperative azoospermia. The mean age was 36 years, and the location of the cryptorchid testes varied, with 66 men showing intra-abdominal testes, 33 men with inguinal testes, eight with one inguinal and one intra-abdominal, and 58 unspecified. A total of 140 men underwent orchidopexy (106 bilateral, 34 unilateral) [10]. Another study by Zhang et al. was reviewed, which analyzed the fertility benefits of orchidopexy in bilateral cryptorchidism in 28 participants, all of whom showed absolute preoperative azoospermia. Of the 28, 22 participated in the postoperative fertility follow-up. Three of these 22 showed spermatozoa in their ejaculate [14].

During fetal development, the testes begin developing in the abdomen. They gradually descend the inguinal canal and finally reach their destination in the scrotum. Cryptorchidism, or undescended testes, is the most common genital abnormality among newborn boys and is present in 1-3% of term males [1]. The prevalence increases to 30% in premature newborn males [4]. The majority of cryptorchid testes spontaneously resolve by six months of life, but those that do not spontaneously resolve, are typically detected during infancy and should be surgically corrected by one year of life [2]. Occasionally, cases go undetected, and males reach adulthood with cryptorchid testes. The prevalence of cryptorchidism in adulthood is low at 0.8-1% [10]. Bilateral cryptorchidism is even less prevalent, accounting for only 30% of all adult cryptorchidism cases [10].

The association between cryptorchidism and male infertility is well documented, although not a major concern in our case [1]. In one report, 10% of infertile adult men had a history of cryptorchidism, and azoospermia was found in 13% of individuals with unilateral cryptorchidism, with azoospermia increasing in 89% in individuals with bilateral cryptorchidism [1]. In cryptorchid testes, spermatogenesis decreases over time, with irreversible damage after the age of two years [15]. In one study, 52 undescended testes, from patients with a mean age of 26 years, were histologically examined revealing only one testicle with normal spermatogenesis [8,15]. A case report by Cito et al. examined a 33-year-old patient with bilateral cryptorchidism and azoospermia. The patient opted for bilateral orchiectomy and Cito’s group explored the possibility of testicular sperm extraction (TESE) to cryopreserve any mature spermatogonia from the resected testes. Unfortunately, only immature sperm cells were found, indicating Sertoli cell-only syndrome. Although the chances of retrieving viable sperm in the setting of orchiectomy for bilateral cryptorchidism are exceedingly low, the authors still stressed that TESE is a good option with no negative impacts on the patient’s health [11]. However, regarding orchidopexy, one study showed that in a group of 22 azoospermic men with inguinal bilateral cryptorchidism, 10 (45.5%) showed viable sperm following a delayed TESE (median 10 months post-orchidopexy). In fact, six of those men experienced successful paternity [10]. Of course, when comparing these two treatment modalities, i.e., orchiectomy versus orchidopexy, malignancy risk is of utmost concern.

The correlation between cryptorchism and testicular cancer is another well-accepted and concerning association [1]. Some studies questioned the utility of testicular biopsy at the time of surgical treatment due to the increased risk of abnormal testicular germ cell synthesis, seen as early as six months of the testis in its cryptorchid position [2]. Studies have shown a direct correlation between the time in which testes are in their cryptorchid position and the incidence of testicular germ cell tumors, most commonly seminomas, with as high as a four-fold increased risk of malignancy [10]. In addition, the risk of germ cell tumors increases even further if the testes are located intra-abdominally [12]. Pettersson et al. found that patients who underwent corrective surgery for cryptorchidism before the age of 13 had an incidence rate of testicular cancer of 2.23%, while patients who underwent corrective surgery after the age of 13 had an incidence rate of 5.40% [16].

Although the risk of testicular cancer is decreased with earlier correction of UDTs, performing an orchiopexy does not completely eliminate the risk of cancer [15]. For this reason, as well as the low fertility potential in a UDT, orchiectomy may be the superior method of intervention for patients with cryptorchidism, especially in adult patients where the testes were in the cryptorchid position for a longer duration. In conjunction with orchiectomy, androgen replacement therapy and evaluation for adjuvant chemotherapy may be appropriate [13]. In patients with a single testicle or those with bilateral cryptorchidism, where there is not a contralateral scrotal testicle, orchiectomy may not be the best option. Of note, the necessity of hormonal replacement with orchiectomy was the leading factor in the decision for our patient to undergo orchiopexy. After an honest and elaborate conversation with the patient and the patient’s parents, it was essential to eliminate the possibility of needing lifelong hormonal replacement. Due to distance from healthcare and the patient’s comorbidities, it would have been more detrimental in cost and quality of life to require lifelong hormonal replacement than infertility and increased follow-up. In cases such as ours, orchiopexy may be preferred with consideration to the fact that these patients are at an increased risk of infertility and testicular cancer. As such, these patients should potentially have a biopsy at the time of surgery to assess early neoplastic changes, as well as receive close, long-term follow-up postoperatively.

Finally, it is worth noting that one of the key factors that likely contributed to this particular patient’s delayed presentation was limited access to healthcare in his region of rural Kentucky. This detail cannot be ignored, considering the dramatically increased malignancy and infertility risk associated with carrying UDTs so far into adulthood. A 2012 study by Wilson et al. examined the root causes of healthcare disparities in low-income regions of the United States, including rural Appalachia [17]. A total of 921 rural residents were sampled on their opinions on local healthcare access. The study concluded that members of these communities, regardless of empirical metrics on the healthcare facilities available to them, did not perceive their access to healthcare to be an issue. This perception is likely to be a major contributor to healthcare inequality and should be more thoroughly explored.

## Conclusions

Cryptorchidism is one of the most common genital abnormalities among male newborns. Current recommendations are that cryptorchid testes be surgically corrected by age one to two to reduce the risk of infertility and testicular cancer. For postpubertal children, the American Urological Association guidelines recommend orchiectomy, again, due to the cancer risk. Occasionally, cryptorchid testes remain undiagnosed during the pediatric period, causing patients to present in adulthood. Due to its rarity in the adult population and lack of published data, no society guidelines currently exist regarding the management, surgical correction, and postoperative follow-up of adult cryptorchidism. Our literature review found that a large number of adult males with cryptorchidism had either pre-neoplastic or neoplastic testes at the time of surgical intervention, suggesting orchiectomy is the safer management option. However, in select patients and with appropriate counseling, orchiopexy may be a viable option to prevent the need for long-term hormone replacement. These adult patients at the time of orchiopexy should have a testicular biopsy, as well as close long-term follow-up to screen for testicular cancer. However, further data is needed to support this.
